# Theta-Range Oscillations in Stress-Induced Mental Disorders as an Oscillotherapeutic Target

**DOI:** 10.3389/fnbeh.2021.698753

**Published:** 2021-06-09

**Authors:** Toya Okonogi, Takuya Sasaki

**Affiliations:** Laboratory of Chemical Pharmacology, Graduate School of Pharmaceutical Sciences, The University of Tokyo, Tokyo, Japan

**Keywords:** oscillations, emotion, depression, anxiety, hippocampus, amygdala, prefrontal cortex

## Abstract

Emotional behavior and psychological disorders are expressed through coordinated interactions across multiple brain regions. Brain electrophysiological signals are composed of diverse neuronal oscillations, representing cell-level to region-level neuronal activity patterns, and serve as a biomarker of mental disorders. Here, we review recent observations from rodents demonstrating how neuronal oscillations in the hippocampus, amygdala, and prefrontal cortex are engaged in emotional behavior and altered by psychiatric changes such as anxiety and depression. In particular, we focus mainly on theta-range (4–12 Hz) oscillations, including several distinct oscillations in this frequency range. We then discuss therapeutic possibilities related to controlling such mental disease-related neuronal oscillations to ameliorate psychiatric symptoms and disorders in rodents and humans.

## Introduction

The accumulation of mental stress loads is a primary risk factor for psychiatric disorders such as major depressive disorder (MDD), anxiety disorders, and posttraumatic stress disorder (PTSD) (Yehuda and LeDoux, 2007; Arnsten, 2015). A number of studies have revealed that brain regions such as the medial prefrontal cortex (mPFC), cingulate cortex, amygdala (AMY), hippocampus (HPC), and hypothalamus play crucial roles in the regulation of affective and visceral functions and undergo marked changes in their activity caused by stress-induced mental disease (Greicius et al., 2007; Sheline et al., 2010; Nugent et al., 2015; Tovote et al., 2015; Drysdale et al., 2017). In particular, the HPC-PFC-AMY circuit is a core network formed by long-range projections (Caliskan and Stork, 2019) in which the ventral HPC (vHPC) and the mPFC transfer sensory and contextual information to the basolateral amygdala (BLA) (Orsini et al., 2011; Ciocchi et al., 2015) and the BLA, in turn, transfers information of negative valence back to the mPFC and vHPC (Ishikawa and Nakamura, 2003; Senn et al., 2014; Kim et al., 2016; Burgos-Robles et al., 2017).

To date, a key technique to understand the basic neuronal mechanisms and devise therapeutic strategies based on pathophysiology is the recording of electrophysiological signals that represent brain activity patterns and provide great temporal resolution at the millisecond scale. The mammalian forebrain generates extracellular field potentials containing a mixture of diverse neural oscillations at frequency bands ranging from 0.1 to 250 Hz (in health) and up to 500 Hz (in disease) that show dynamic changes associated with arousal levels, emotional valence, and memory performance (Buzsaki and Draguhn, 2004; Buzsaki et al., 2012; Buzsaki and Watson, 2012). In addition to representing the activity patterns of individual brain areas, electrical signals from multiple brain regions are sensitive to changes in their functional connectivity defined as their correlational power changes and coherence. A number of clinical studies have reported altered patterns of electroencephalogram (EEG) oscillatory signals in depressed patients, such as altered power and functional coupling in the alpha (8–13 Hz) and gamma (30–100 Hz) bands in the frontal cortex (Jesulola et al., 2015; Fitzgerald and Watson, 2018; Grunewald et al., 2018). Similarly, in PTSD patients, resting electrical signals in the PFC show decreased alpha power-mediated inhibition and increased gamma power, suggesting hypofunction in the PFC (Huang et al., 2014; Clancy et al., 2017). Accumulated evidence from these studies suggests that brain field potential signals serve as a physiological sign of mood disorders (Iosifescu, 2011; Baskaran et al., 2012; Buzsaki and Watson, 2012; Fitzgerald and Watson, 2018).

On the other hand, at microscopic levels, a number of studies from animal models to human patients have demonstrated stress-related molecular and cellular mechanisms that could lead to psychiatric disorders (Krishnan and Nestler, 2008; Arnsten, 2015; McEwen et al., 2015). However, it remains largely unknown how these mechanisms are integrated in the expression of psychiatric symptoms and behavioral phenotypes. The necessity to bridge the gap between these insights also highlights the importance of electrical field signals as a measure to estimate neuronal network-level dynamics. In particular, animal experiments allow us to directly measure local field potential (LFP) signals from target brain regions with high signal-to-noise ratios and compare how their oscillatory patterns dynamically change with emotional behavior in both health and disease. Such basic non-clinical experiments are crucial for devising novel therapeutic strategies, including drug discovery and timed interventions on brain activity, which have been termed oscillotherapeutics (Takeuchi and Berenyi, 2020).

This paper introduces recent techniques to measure brain LFP signals from freely moving rodents, summarizes recent reports showing anxiety- and fear-related changes in LFP patterns, especially focusing on the HPC-PFC-AMY circuit, observed in non-pathological animals, and then describes how LFP signals from these brain regions are affected by stress accumulation. Finally, we discuss potential therapeutic strategies to ameliorate stress-induced psychiatric disorders based on oscillatory LFP patterns.

## Methods for Oscillotherapeutic Studies Using Rodents

### Multisite Recordings of Local Field Potentials (LFPs) in Freely Moving Rodents

**FIGURE 1:**
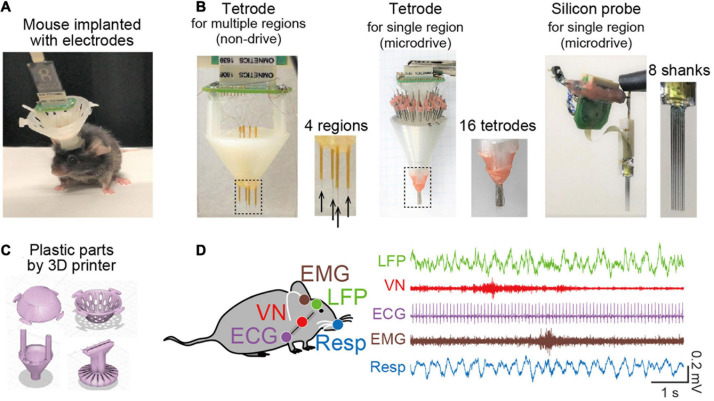
Recent recording methods to study brain oscillations in rodents. **(A)** A picture of a mouse implanted with an electrode assembly. **(B)** Typical electrode assemblies with multiple recording sites. (Left) Tetrode arrays to target several separated brain regions. The dotted region is magnified in the right panel, showing the electrode tips (indicated by arrows). (Middle) A microdrive with multiple tetrode arrays to record spike patterns of neurons in a target region. (Right) A microdrive with 8-shanks silicon probes to record spike patterns of neurons in a target region. **(C)** Typical CAD illustrations of plastic parts created by a 3D printer. **(D)** Simultaneous electrophysiological recordings of a brain LFP signal, a vagus nerve (VN) signal, an ECG signal, an EMG signal, and a Respiration (Resp) signal.

A key experimental technique related to oscillotherapeutics in rodent research is chronic electrophysiological recordings of extracellular signals representing collective oscillatory field potentials from neuronal populations and spike patterns from individual neurons in freely moving animals (Figure 1A). A number of studies have utilized various types of recording electrodes, such as tetrode arrays and silicon probes (Figure 1B), that enable multisite (tens or hundreds of sites) recordings from target brain regions. These electrodes are chronically implantable for several months and are flexibly movable to adjust the depth of electrodes with micrometer precision in the brain tissue after implantation using microdrives. Recent advancements in 3D printer technology make it easy to customize plastic parts to accommodate these electrodes into a microdrive (Figure 1C). For example, we recently created a recording device to cover wide ranges of cortical regions from anterior to posterior and from medial and lateral parts in rodents (Figure 1B, left) (Konno et al., 2019; Nakayama et al., 2019). Conveniently, most of the CAD files designed by developers for multichannel recordings are now available from cloud-based repositories, such as Mendeley data, and laboratory websites, which enables researchers to freely create these devices. Furthermore, wireless recording systems are recently available (Zuo et al., 2012; Martinez et al., 2018; Iturra-Mena et al., 2019), which are especially useful for stress research because they reduce the physical stress of animals.

In addition to brain electrophysiological recordings, we conceived a recording approach in which a multichannel recording device extends to the collection of bioelectrical signals from peripheral organs, such as electrocardiogram (ECG) signals, electromyogram (EMG) signals (Okada et al., 2016; Okonogi et al., 2018; Shikano et al., 2018), olfactory bulb respiratory (Resp) signals (Kuga et al., 2019), and vagus nerve (VN) signals (Shikano et al., 2019) (Figure 1D), all of which can be captured by a single recording device. This recording method is useful for precisely monitoring signals representing changes in peripheral organ activity related to emotion, stress, and mental disorders, in addition to simple behavioral phenotypes.

### Realtime Manipulation of Local Field Potentials

**FIGURE 2:**
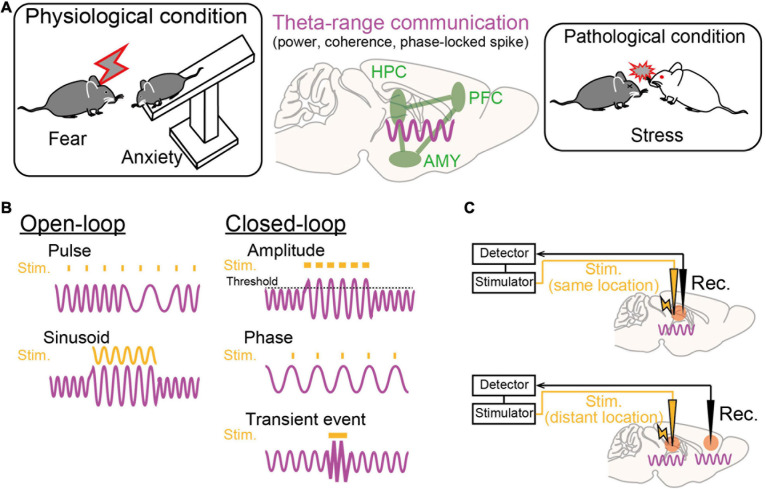
Theta-range oscillations as a target of oscillotherapeutics. **(A)** Recent studies suggest that theta-range (4–12 Hz) oscillations in the HPC-PFC-AMY circuit are crucial for emotionalbehavior and susceptibility to stress. **(B)** Examples of stimulation patterns (orange) upon brain oscillatory signals (magenta) in the open-loop and closed-loop systems. **(C)** (Top) Closed-loop stimulation is applied to the brain region where a target brain signal is recorded. (Bottom) Closed-loop stimulation is applied to a brain region that is different from the brain region where a target signal is recorded.

Electrophysiological recordings, compared with imaging techniques, provide higher temporal resolution at millisecond timescales, allowing real-time detection of electrical signals and preciselytimed interventions involving neuronal activity immediately upon theemergence of target features in the signals (e.g., signal amplitude, phase, and spikes), a so-called closed-loop neurostimulation system (Figures 2B,C). On-demand stimulation protocols based on this system enable a high-quality physiological experimental design for both basic and pathological studies. As an example of targeting transient brain signals, time-specific stimulation during seizure events can inhibit subsequent seizure-like behavior in epilepsy animal models (Berenyi et al., 2012; Takeuchi et al., 2021). At frequencies lower than 10 Hz, phase-targeting stimulation (e.g., peaks and troughs of a given oscillation) is an effective technique to test their contributions to brain functions. For instance, theta (4–12 Hz) phase-specific manipulations of neuronal activity in the hippocampus and the subthalamic nucleus have been shown to induce memory enhancement and parkinsonian symptoms, respectively (Siegle and Wilson, 2014; Cordon et al., 2018). Additional details of applications to studies of emotion and psychiatric disease are described later.

## Theta-Range Oscillations in the HPC-PFC-Amy Circuit Related to Emotional Behavior

Dysregulation of emotions and increased anxiety are crucial hallmarks of stress-induced mental disorders in both animal models and human patients. Regarding rodent studies, behavioral paradigms have been established to subjectively estimate the levels of emotional valence and anxiety. From these behavioral experiments, a number of studies with genetic and pharmacological approaches have suggested that the HPC-PFC-AMY regions is a hub network related to fear and anxiety-like behavior (as reviewed by Tovote et al., 2015). In particular, electrophysiological studies have indicated the importance of theta-range (4–12 Hz, including multiple distinct oscillations) LFP signals in the HPC-PFC-AMY circuit related to emotional behavior as potential substrates for temporal circuit coordination and long-term plasticity in neuronal networks (Figure 2A; as reviewed by Caliskan and Stork, 2019). Experimentally, such low-frequency signals are a good model for a closed-loop system (e.g., phase-targeting stimulation) to test their causal roles in behavior and to associate neuronal activity patterns with single spike levels. Here, we focus on several major findings of theta-range LFP oscillations in the HPC-PFC-AMY regions observed in non-pathological animals, which are subsequently discussed in later chapters from the perspective of pathology. Details regarding the involvement of the other oscillations and the other brain regions are beyond the scope of this paper.

### Fear

Fear is an adaptive component of transient responses to internal and external aversive events such as potentially threatening stimuli. Fear conditioning tests are often used to assess rodents’ learned fear, in which a conditioned stimulus (e.g., auditory stimulus) is paired with an aversive unconditioned stimulus. During an acquisition phase of fear conditioning, the mPFC-BLA circuit has been shown to increase 4-Hz LFP power (Karalis et al., 2016; Davis et al., 2017). During REM sleep periods after fear conditioning, the vHPC-mPFC-BLA circuit exhibits long-lasting enhancement of theta (4–12 Hz) power and interregional theta synchrony for hours (Popa et al., 2010; Ognjanovski et al., 2014; Totty et al., 2017), possibly serving as a substrate to consolidate fear memories (Boyce et al., 2016). During retrieval phases where the same conditioned stimulus is applied, similar tendencies of the two types of LFP oscillations are detected; increased mPFC-BLA 4-Hz (Dejean et al., 2016; Karalis et al., 2016; Ozawa et al., 2020) and HPC-mPFC-BLA theta (4–12 Hz) oscillations (Seidenbecher et al., 2003; Likhtik et al., 2014; Stujenske et al., 2014), each of which entrain oscillatory spike patterns of cell ensembles in this frequency band. Note that Karalis et al. (2016) suggested that the mPFC-BLA 4-Hz oscillation is distinct from the theta oscillations with higher frequency ranges as they are generated through different mechanisms. First, medial septum inactivation selectively eliminated the theta oscillations, possibly via the HPC, while the mPFC 4-Hz oscillation remained intact, which suggests that medial septum is an upstream brain region providing theta-locked inputs to the hippocampus (Buzsaki, 2002) but not 4 Hz-locked inputs to the mPFC. Second, HPC theta oscillations appear to represent atropine-sensitive type 2 theta oscillations as they are specific to periods of immobility (Seidenbecher et al., 2003) and responses to danger (e.g., predator odor) (Mikulovic et al., 2018). This insight suggests that HPC-mPFC-BLA theta oscillations depend on cholinergic inputs, possibly through the medial septum. Third, dmPFC interneurons exhibit spike patterns phase-locked to 4-Hz oscillations (Karalis et al., 2016) and selective activation of dmPFC parvalbumin (PV)-expressing interneurons replicates dmPFC 4-Hz oscillation (Dejean et al., 2016). These results suggest that dmPFC 4-Hz oscillation is intrinsically induced from the inhibitory neuronal circuit in the dmPFC.

Manipulation of the mPFC 4-Hz oscillations is useful to test their causal roles in fear expression and retrieval. Karalis et al. (2016) has demonstrated that optogenetic induction of dmPFC 4-Hz oscillations drives conditioned freezing and Dejean et al. (2016) has demonstrated that inhibition of dmPFC principal neurons in the descending phase of the oscillation increased conditioned freezing. These results suggest the sufficiency of dmPFC 4-Hz oscillations in the induction of learned freezing behavior.

Conditioned fear memories are extinguished by repeated presentations of the conditioned stimulus alone without presentation of an unconditioned stimulus, which is termed extinction learning. Consistent with increased theta oscillations during freezing throughout the phases of fear conditioning, theta coupling across the HPC-mPFC-BLA regions declined as animals underwent extinction learning, whereas it recurred during extinction recall (Lesting et al., 2011). Davis et al. (2017) demonstrated that increased power of a 3–6-Hz BLA oscillation, possibly classified as a 4-Hz oscillation, induced postextinction fear memory retrieval. In contrast, outcompeting this BLA oscillation by chemogenetic inhibition of BLA parvalbumin-positive interneurons (Davis et al., 2017) or 8-Hz sinusoidal stimulation (Ozawa et al., 2020) could inhibit the recurrence of fear behavior following extinction learning, which suggests the necessity of a 4-Hz oscillation in fear extinction recall and/or the induction of conditioned freezing.

### Anxiety

Anxiety is an emotional state characterized by an unpleasant state with heightened awareness even without actual exposure to danger. Anxiety-like behavior in rodents is generally assessed in an elevated plus maze (EPM) test or an open field (OF) test. Through these behavioral tests, a number of studies have suggested that the expression of both fear and anxiety is mediated by the HPC-PFC-AMY circuit, possibly through overlapping neuronal mechanisms (Tovote et al., 2015). As a typical example, mice lacking serotonin 1A receptors showing higher anxiety-like behavior in an EPM test exhibited larger increases in HPC theta (4–12 Hz) power (Gordon et al., 2005). In contrast, treatment with anxiolytics, such as serotonin 1A receptor agonists and benzodiazepines, has been shown to exhibit decreases in HPC theta oscillations (Zhu and McNaughton, 1995). Adhikari et al. (2010) recorded LFP signals simultaneously from the mPFC and vHPC in mice engaging in an EPM test. They showed that theta-frequency communication between the mPFC and vHPC was specifically augmented under anxiogenic environments in an EPM and an OF and correlated with behavioral performance (Adhikari et al., 2010). At a single-neuron level, mPFC neuronal firing patterns were precisely entrained by vHPC theta oscillations (Adhikari et al., 2011), which were related to anxiogenic behavior in an EPM. Together with the fact that the vHPC neurons preferentially send anxiety-related information to the mPFC (Ciocchi et al., 2015), it is conceivable that vHPC theta oscillations lead to mPFC theta-locked spiking activity, and such theta-locked mPFC neuronal activity is crucial for the expression of anxiety. This idea was further supported by the observation that artificial oscillatory activation of mPFC-projecting vHPC neurons at a theta (8-Hz) frequency increased anxiety-like behavior in an EPM test (Padilla-Coreano et al., 2019). In addition, Likhtik et al. (2014) demonstrated that mPFC theta oscillations precede BLA spiking activity and that coherence and power changes at the theta band in the mPFC-BLA circuit predicted stay time in anxiogenic environments. These results all suggest that interregional coordination of neuronal population activity in theta bands in the vHPC-mPFC-BLA circuit is crucial for the expression of anxiogenic behavior. Together with the observations of fear-related theta-range oscillations, anxiogenic behavior and fear behavior may be expressed partly through a common mechanism: theta-range power increases as a means for enhanced synchronization and entrainment of the HPC-PFC-AMY circuit.

## Oscillations in the HPC-PFC-Amy Circuit Related to Stress-Induced Psychiatric Disorders

The findings of theta-range oscillations in the HPC-PFC-AMY circuitfrom non-pathological animals related to fear and anxiety-like behavior suggest that stress susceptibility and stress-induced dysregulation of emotion may be due to altered interplay based on these oscillatory communications. In rodents, behavioral and physiological changes reminiscent of depressive symptoms are induced by repeated exposures to chronic social defeat stress in which a mouse is defeated by a larger animal; this procedure has been utilized as an excellent murine model with etiological, predictive, discriminative and face validity (Berton et al., 2006; Golden et al., 2011; Abe et al., 2019). Socially defeated mice with impaired extinction learning, possibly replicating a PTSD symptom, showed increased theta (4–8 Hz) synchronization between the PFC and AMY (Narayanan et al., 2011). In addition, defeated mice with depressive-like behavior showed increased power of a PFC 2–7-Hz oscillation during interactions with an aggressor mouse (Kumar et al., 2014), which entrained beta (14–23 Hz) coherence between the AMY and ventral tegmental area (VTA) (Hultman et al., 2016). These stress-sensitive changes in oscillatory patterns possibly represented an increase in the 4-Hz or theta oscillations based on their frequency bands and common properties. Consistently, increased theta (4–12 Hz) power in the vHPC-mPFC-BLA circuit has also been reported in a chronic unpredictable stress model (Jacinto et al., 2013; Oliveira et al., 2013). Taken together, these findings suggest that stressed animals appear to exacerbate aversive emotion-related oscillations in the HPC-PFC-AMY circuit that originally operated in non-pathological conditions.

Notably, the power of this 2–7-Hz PFC oscillation differs across individual animals even before stress exposure, and these differences can predict the manifestation of subsequent stress-induced depression-like behavior (Kumar et al., 2014). This finding suggested that LFP signals can serve as a predictive factor of vulnerability to mental stress in individual animals. Recently, this idea of predictive stress vulnerability has been expanded to multiple brain regions, including the nucleus accumbens (NAc) and VTA, in addition to the HPC-PFC-AMY circuit. An elegant study with a machine learning algorithm by Hultman et al. (2018) identified several patterns of prestress LFP power and coherence in frequency bands ranging from 1 to 50 Hz across these brain regions and termed these patterns electome factors. These factors differentiate early life-associated stress vulnerability and stress susceptibility that can be reversed by antidepressants. Our group applied a similar strategy to LFP signals recorded from multiple cortical areas and demonstrated that rats with lower theta power and higher delta power correlations across the cortical regions before stress exposure were more likely to exhibit irregular heartbeat signals after stress load (Nakayama et al., 2019). In the future, these new types of studies with multivariate statistics and machine learning algorithms on large-scale physiological datasets are expected to reveal how the core region, i.e., the HPC-PFC-AMY circuit, and neuromodulatory regions, such as the NAc and VTA, functionally interact with each other. These new approaches are expected to provide a more comprehensive entire picture of functional organizations of brain networks that have not been defined by existing statistics with limited dimensions.

## Time-Targeted Interventions Toward Oscillotherapeutics for Psychiatric Disorders

While LFP oscillatory signals in the HPC-PFC-AMY circuit related to stress-induced symptoms are beginning to be revealed (Figure 2A), they remain correlative and leave open questions as to whether they are causal factors for the pathogenesis of mental disorders. Addressing these issues will help identify true therapeutic targets of endogenous oscillatory signals in the development of oscillotherapeutics. Ideal research strategies are to selectively manipulate target ongoing oscillatory signals using open-loop or closed-loop systems and test their phenotypic effects. In open-loop interventions, external stimulation with sinusoidal waveforms, mimicking oscillatory signals, or pulse trains is applied without feedback from biological oscillatory signals (Figure 2B, left). In closed-loop interventions, stimulation is applied with the appearance of target brain signals, enabling on-demand stimulation with reference to brain states while avoiding out-of-target overstimulation (Figure 2B, right). Target signal variables to be detected are transient neuronal events with certain amplitudes, such as ictal seizure events, or the phase and amplitude of specific continuous oscillations, such as theta oscillations. Signals to be manipulated are the signals detected at the recording sites or signals generated at distant areas, such as upstream or downstream brain regions, depending on the locations of stimulation (Figure 2C). This chapter summarizes recent studies employing these experimental challenges in rodents and discusses the possibility of further clinical applications. Other therapeutic strategies using pharmacological, behavioral, and psychological methods to alter brain oscillations (e.g., Leuchter et al., 2015) are beyond the scope of this paper.

### Animal Models

Transient neuronal events within a short time window (hundreds ofmilliseconds) are widely utilized target signals for closed-loopsystems. For instance, transcranial and intracranial closed-loopstimulation at the time of detection of large amplitude andhigh-frequency cortical seizure events have been shown to suppresssubsequent seizures in epilepsy animal models (Berenyi et al., 2012; Takeuchi et al., 2021). This technique has also been applied to LFP oscillations under non-pathological conditions to test their causal roles in memory processing. For example, closed-loop amplification and disruption of hippocampal ripples (150–250 Hz), which represent transient neuronal synchronization within a short time window (∼100 ms), can improve (Fernandez-Ruiz et al., 2019) and inhibit (Jadhav et al., 2012; Oliva et al., 2020; Igata et al., 2021), respectively, subsequent memory processing. While targeting this type of transient signal is an excellent means to manipulate large short neuronal events, it appears inappropriate for modulating emotional states, as brain signals underlying emotion and stress-induced disease are much longer and oscillate in lower frequency bands, as described above.

Given the importance of theta-range (4–12 Hz) oscillations in the HPC-PFC-AMY circuit in emotional behavior and stress-induced psychiatric disorders, manipulation of neuronal activity that impact these theta-range oscillations might be effective in ameliorating psychiatric symptoms. Using open-loop systems, theta-range oscillations can be exogenously induced by injecting sinusoidal stimulation at the corresponding frequencies. In particular, optogenetic photostimulation is appropriate for creating sinusoidal stimulus patterns with particular rise and decay amplitudes to manipulate specific types of neurons. Padilla-Coreano et al. (2019) demonstrated that selective activation of mPFC-projecting neurons in the vHPC by optogenetic photostimulation with a theta (8-Hz) sinusoidal pattern evoked open arm avoidance in an EPM, suggesting increased anxiety. Interestingly, this effect was not observed when an 8-Hz pulsatile stimulation pattern was applied, suggesting the importance of oscillatory neuronal activity. Karalis et al. (2016) demonstrated that modulation of mPFC parvalbumin-expressing (PV) interneurons by 4-Hz, but not 8-Hz, sinusoidal photostimulation induced synchronized mPFC and BLA spikes and contextual fear behavior, suggesting a causal role of 4-Hz oscillations in the expression of aversive memories. Furthermore, using a similar strategy, Ozawa et al. (2020) showed that rhythmic stimulation of BLA PV interneurons with a sinusoidal waveform of light at 4 Hz or 8 Hz augmented or suppressed freezing behavior, respectively, specifically after a postextinction learning trial, demonstrating the bidirectional modulation of extinction memories by BLA PV interneurons depending on different oscillatory frequencies. In addition, they suggested that extinction learning with suppression of conditioned fear cell ensembles in the BLA-mPFC circuit was mediated by BLA PV interneurons via enhancement of a BLA 8-Hz (6–12 Hz) oscillation that interfered with a BLA-mPFC 4-Hz (3–6 Hz) oscillation for fear expression (Davis et al., 2017). Further causal relationships can be tested by a closed-loop system with higher temporal resolution in which stimulation is applied at a specific phase of each cycle of oscillations. Dejean et al. (2016) demonstrated that phase-targeting optogenetic activation of mPFC PV interneurons in the ascending or descending phases of mPFC 4-Hz oscillations decreases and increases conditioned freezing behavior, respectively, suggesting the importance of phase-specific mPFC cell ensemble activity for bidirectional control of fear behavior. Using similar experimental strategies, increased anxiety and impaired fear extinction by chronic stress might be restored by adjusting the intensity of theta-range oscillations and phase-specific modulation of neuronal activity on theta-range oscillations in the HPC-PFC-AMY circuit. Similar ideas that target theta-range oscillations have been recently applied to other animal models of Parkinson’s disease and Alzheimer’s disease (Cordon et al., 2018; Senova et al., 2018).

For more complex challenges regarding the understanding of functional connectivity and interregional information transfer underlying emotional behavior, manipulations of multiple brain regions at the same or different phases in oscillations may be more useful (Figure 2C), as demonstrated by Ozawa et al. (2020). Finally, animals’ behavioral states (e.g., sleep states and attentional levels) are also crucial factors in interventions of neuronal activity. For example, Boyce et al. (2016) demonstrated that a reduction in HPC theta oscillations specifically during REM sleep eliminated conditioned fear memories, suggesting that applying interventions in the proper behavioral states may be important to maximize their outputs and avoid side effects.

### Application to Clinical Studies

While it is not possible to simply extrapolate clinical applications based on insights from basic animal studies, interventions targeting brain oscillations may also be an effective therapeutic strategy in clinical studies if properly applied. Deep brain stimulation (DBS) is an invasive treatment approach for treatment-resistant depressions applied by electrodes implanted in brain tissue (Mayberg et al., 2005; Scangos et al., 2021). On the other hand, transcranial electric stimulation is a non-invasive approach that is roughly divided into two types depending on stimulus patterns: transcranial direct current stimulation (tDCS) with non-oscillating static currents and transcranial alternating current stimulation (tACS) with oscillating currents. For more focal and intense stimulation, repetitive transcranial magnetic stimulation (rTMS) applies magnetic pulses to induce current flow in the brain. Accumulating clinical evidence suggests that restorations of brain oscillations by these interventions ameliorate pathological symptoms in depressive patients. For instance, rTMS on the frontal cortex increased gamma oscillations in patients showing improved depressive symptoms (Noda et al., 2017), consistent with a pharmacological effect of ketamine on gamma oscillations (Hong et al., 2010). Vagus nerve stimulation (VNS) is another intervention approach for treatment-resistant depression (Moeller et al., 2019). This method directly activates subcortical arousal-promoting nuclei through stimulation of the vagus nerve. Recently, a non-invasive transcutaneous auricular vagus nerve stimulation (taVNS) method has been developed (Hein et al., 2013; Rong et al., 2016; Trevizol et al., 2016), which can alter alpha EEG oscillations (Sharon et al., 2021).

Compared with simple pulsatile stimulation such as tDCS, resonance approaches using oscillatory stimulus patterns such as tACS may be more effective in entraining brain oscillations at the corresponding or other specific frequency bands [for more detail, see Hanslmayr et al. (2019)]. For instance, in healthy human subjects, intense tACS with a 1-Hz sinusoidal wave has been shown to induce alpha-band parietal cortical activity (Voroslakos et al., 2018). In older adults, tACS over the frontotemporal regions improves memory performance and cognitive functions (Reinhart and Nguyen, 2019). More specifically, a closed-loop system using DBS may be a promising strategy, in which brief stimulation is delivered in response to ongoing changes in the brain states of patients (Scangos and Ross, 2018). In addition, biofeedback methods in which individuals are provided with real-time information on brain activity have been proposed as another means to modify EEG signals and improve psychiatric states. For instance, restoring frontal alpha symmetry by biofeedback signals has been shown to reduce negative affect in human subjects that learned conscious control of their own alpha asymmetry signals (Mennella et al., 2017).

## Conclusion and Future Perspective

Recent advancements in electrophysiological recording techniques, optogenetic tools, and analytical methods such as machine learning algorithms unveiled emotion-related and stress-induced oscillations for complex coordination of multiple brain regions. This review specifically focused on theta-range oscillations, as a key oscillation, in the HPC-PFC-AMY circuit in rodents. Precisely timed interventions based on these oscillations by open-loop and closed-loop systems enable us to test a causal role of these target oscillatory signals in the expression of psychiatric symptoms. In the future, in addition to observing changes in behavioral phenotypes, it will be interesting to see how emotional memory-encoding and oscillatory phase-locked neuronal ensembles are recruited at single-neuron levels in response to these interventions. While the same experimental strategies may not be directly extrapolated to clinical studies, the ideas of interventions are adopted as effective therapeutic strategies for stress-induced mental illness. In both animal and clinical studies, a crucial issue is that there are a large number of combinations of technical parameters (e.g., stimulation regions, type, intensity, timing, and frequency band) for interventions. In addition, as the level of impairments in these brain signals and pathological symptoms considerably vary across individuals, interventions need to be ideally personalized based on their conditions, as previously demonstrated (Reinhart and Nguyen, 2019; Grover et al., 2021). Further oscillotherapeutic studies from both basic and clinical experiments are expected to identify more appropriate intervention strategies based on a precise understanding of cellular and circuit dynamics.

## Author Contributions

TO and TS prepared all the figures and wrote the main manuscript text. Both authors contributed to the article and approved the submitted version.

## Conflict of Interest

The authors declare that the research was conducted in the absence of any commercial or financial relationships that could be construed as a potential conflict of interest.

## Abbreviations

AMY: amygdala
BLA: basolateral amygdala
LFP: local field potential
mPFC: medial prefrontal cortex
PTSD: posttraumatic stress disorder
vHPC: ventral hippocampus.
